# Explainable Machine Learning for Tower-Radar Monitoring of Wind Turbine Blades: Fine-Grained Blade Recognition Under Changing Operational Conditions

**DOI:** 10.3390/s26041083

**Published:** 2026-02-07

**Authors:** Sercan Alipek, Christian Kexel, Jochen Moll

**Affiliations:** 1 Department of Mechanical Engineering, University of Siegen, Paul-Bonatz-Straße 9-11, 57076 Siegen, Germany; jochen.moll@uni-siegen.de; 2 Verein für Datenwissenschaften (VEFDAWI), 60326 Frankfurt am Main, Germany

**Keywords:** tower-radar monitoring, wind turbine blade classification, explainable AI, deep learning, radar sensor system, wind energy

## Abstract

This paper evaluates a data-driven classification approach of operational wind turbine blades based on consecutive tower-radar measurements that are each compressed in a two-dimensional slow-time to range representation (radargram). Like many real-world machine learning systems, installed tower-radar systems face some key challenges: (i) transferability to new operational contexts, (ii) impediments due to evolving environmental and operational conditions (EOCs), and (iii) limited explainability of their deep neural decisions. These challenges are addressed here with a set of structured machine learning studies. The unique field data comes from a sensor box equipped with a frequency-modulated continuous wave (FMCW) radar (33.4–36 GHz frequency range). Relevant parts of the radargram that contribute to a decision of the used convolutional neural networks were identified by a class-sensitive visualization technique named GuidedGradCAM (Guided Gradient-weighted Class Activation Mapping). The following main contributions are provided to the field of tower-radar monitoring (TRM) in the context of wind energy applications: (i) every individual rotor blade holds a number of characteristic structural features revealed by the radar sensor, which can be used to discriminate rotor blades from the same turbine via neural networks; (ii) those unique features are not agnostic to changing EOCs; and (iii) pixel-level distortions reveal the necessity of low-level information for a precise rotor blade classification.

## 1. Introduction

### 1.1. Motivation for Explainable Machine Learning

Technologies in the realm of machine learning (ML) and artificial intelligence (AI) continue to evolve rapidly and will contribute to progress in various industries. Enhancing predictions and thereby optimizing operations can provide economic advantages as well as socially or environmentally beneficial outcomes, e.g., safety or resilience of energy generation and respective networks, the conservation of biodiversity and ecosystems, as well as climate change mitigation or adaptation. ML, therefore, can substantially serve the public interest. Tower-radar monitoring (TRM) for smart wind turbines represents such an envisioned application portfolio [1,2,3].

In general, power plants supply societies around the world with much-needed energy. High-risk ML applications with respect to such critical infrastructure encompass ML systems intended to be used as safety components in the management and supply of electricity. Here, ML deployment should meet advanced requirements that include transparency of the results or verified reliability against foreseeable deviations from their static training setting. Transparency means that ML gets developed and used in a way that allows appropriate traceability and explainability, as well as laying open the limitations and strengths. The application of those principles should be translated into the design and deployment of ML models, e.g., according to the EU AI Act [4].

In addition to regulatory compliance, explainability nourishes the trust of users and stakeholders in the ML components of intelligent systems. In particular, novel products and services are challenged with gaining acceptance. Explainability enforces productivity, because it remains difficult to identify issues and solutions when models fail, when an understanding about their internal decision-making is lacking [5]. Explainable ML is maturing, and ML gets more and more deployed in real-world applications, and hence a topical issue has become the combination of explainability and robustness analysis.

**Figure 1:**
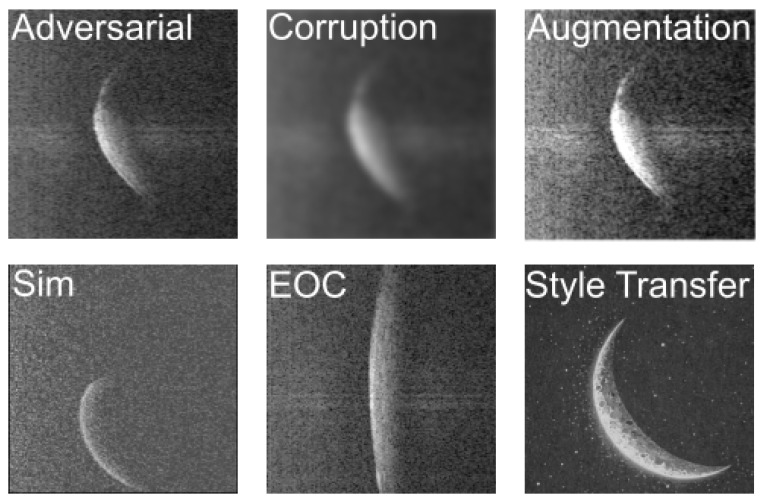
Adjacent concepts from computer vision applied to radargrams: Adversarial example, common corruption (blurriness), data augmentation (contrast or brightness changes), sim2real gap, geometric image transformation (EOCs), style transfer (rotor blade is mistaken for moon crescent). Input image taken from Mälzer et al. [11] and simulation from Kexel [12].

The concept of systematically perturbing a system or process and observing how sensitively outcomes change is a very general idea in science and technology [6]. In particular, an early contribution to explainability in ML has been the introduction of Local Interpretable Model-Agnostic Explanations (LIME). Here, the fundamental insight [7] lies in the use of local perturbations to assess the reliability of the prediction. In this paper, we will use perturbations, e.g., to study visual explanations, among other things. In our case of computer vision, where only the radar-derived image and no context are available at test time, many distinct concepts and image transformations become closely related or cannot be clearly differentiated anymore. Several of those concepts are shown in Figure 1. They include adversarial examples without visible difference to the original image, common corruption (including blurriness), data augmentation techniques (e.g., contrast or brightness changes), sim2real gap [8] between original and synthetic images, geometric image transformation through environmental and operational conditions (EOCs), and style transfer [9,10] where the content of an image is kept but the appearance changes.

### 1.2. Perturbations, Model Robustness and Generalization

Adversarial images [13,14] are specifically designed to mislead models while remaining visually indistinguishable from original images to human observers. These images are created by adding carefully calculated perturbations, such as noise patterns, to exploit vulnerabilities in a model’s decision boundaries. In contrast, data augmentations [15] represent intentional modifications of training data to improve model robustness and generalization. They create plausible variations to learn logical invariances. In particular, data augmentation techniques can be divided into two technical categories [16]: One category comprises collective techniques that coherently alter the geometry, shape, or size of the given image (such as stretching); the other category contains pixel-level transforms, which only manipulate the pixels of an image without changing the overall geometry or size of the image (i.e., such transforms can imitate certain alterations in the measurement process of the radar system).

Moreover, common corruptions represent natural-caused image degradations that occur in real-world settings. They include phenomena like motion blur, noise or compression artifacts. Lastly, transformations of two-dimensional images due to environmental or operational conditions (EOCs) occur when a similar physical scene is captured under different conditions or viewing parameters (geometric angles of the wind turbine tower and rotor blade). These transformations again represent natural variations that occur during field deployments.

This theoretical and motivating perspective connects seemingly different concepts. Understanding these connections helps in holistically developing more robust vision systems that can handle the full spectrum of image variations encountered in the real world.

### 1.3. Related Works and Novelty

The inspection or even monitoring of wind turbine blades can be deployed in many different ways. Typical inspection methods include unmanned aerial vehicles (UAVs) that are remotely controlled to scan the surface of a rotor blade for defects [17]. This approach is accompanied by several challenges and drawbacks compared to ours. First, operating a drone still requires human guidance, which is a costly endeavor when used frequently. Second, this kind of inspection is only possible during downtimes outside the normal operation window of a wind turbine. It is therefore not designed for the continuous monitoring of wind turbines, which requires rapid reactions during service. In addition, it is affected by weather conditions, which further limits the accessibility of a wind turbine and impacts the quality of the measurements.

A recent study on blade surface defect detection has used optical images from a UAV to train a YOLOv10s network for the respective object detection task [18]. Another work applied data fusion on operational wind turbine measurements to improve the segmentation of rotor blades in video data with the help of thermal videos [19]. A general drawback of optical imaging methods is the strong dependency on sunlight exposure. Such monitoring and inspection systems rely on constant ideal lighting because the direction and intensity of the sunlight influence general image quality and can cause distracting shadows or glare.

Radar sensor systems instead offer continuous monitoring of wind turbines much less affected by weather conditions. A recent work performed a fatigue loading experiment on an isolated wind turbine blade with several frequency-modulated continuous wave radar sensors (FMCW) embedded in its fiber mantle [20]. Although directly embedded sensors offer greater potential to detect damage locations with higher accuracy, transferring this setup from the laboratory to real operational wind turbines comes with many technical challenges that have not yet been sufficiently solved. With respect to tower-radar monitoring (TRM), a few previous studies exist. In Moll et al. [21] the feasibility of damage detection with a tower radar in the laboratory was investigated. Moreover, an ML analysis of radar data in the time domain and in the frequency domain was presented separately in another laboratory setting for wind turbine blade materials [22]. Tang et al. [22] successfully distinguished various defect types within a fixed grid of composite cubes using measurements from an FMCW radar with a 24–25.5 GHz band.

Our experimental setup of a tower-mounted radar system that monitors the operational wind turbine blades of the respective tower, to the best of our knowledge, is the first engineering application of its kind.

A detailed description of this setup can be found in Mälzer et al. [11]. In the time of that publication a dataset of 104,000 verified samples made of radargram, label and metadata was collected between 21 October 2022 and 31 March 2023 for one turbine and 59,000 samples for another turbine, collected from 15 December 2022 through 25 February 2023. Mälzer et al. [11] only used one small subset example with 11,500 random samples from the first turbine to validate the general classification potential of the data via a simple convolutional neural network (CNN). This work in comparison includes the complete dataset from the second turbine in Mälzer et al. [11] together with an extended dataset of the first turbine with 276,000 verified samples collected between 21 October 2022 and 23 March 2024. This large dataset was extensively investigated in this paper in terms of transferability between turbines, robustness under unseen environmental and operational conditions and model sensitivity towards physically expected radar signal perturbations accompanied by visual explanations.

Furthermore, in Alipek et al. [23] the ability of a CNN to classify moving rotor blades was discussed when encountering feature drift through changing environmental and operational conditions. It is important to mention that Alipek et al. [23] worked with measurements from a third turbine unrelated to this study, but from the same wind park. The technical setup was equivalent, while two out of three rotor blades of that turbine were differently marked with a microwave absorber foil either facing the radar or the opposite side away from the radar. Alipek et al. [23] analyzed the potential impact of markings on the rotor blade surface by performing a binary anomaly detection task for each individual rotor blade. This task is inherently different from this paper’s efforts that were mentioned before.

An additional context is given by a large-scale study of many common deep learning models on natural images that has investigated how accuracy changes when factors such as the pose of objects are changed [24].

This is analogous to studies focusing on accuracy under common corruptions [25]. Subsequently, the performance under corruptions was studied when various data augmentations are applied [26]. In another study [27], an incremental learning task on synthetic aperture radar data is improved by a LIME-based interpretability metric. Furthermore, the robustness of visual explanations in a medical setting was studied with a focus on the impact of network depth [28]. With respect to adversarial perturbation, the reliability of explainability methods was examined in Galli et al. [29].

Recent work has increasingly addressed interpretability and robustness aspects of learning-based radar signal analysis [30,31,32], as well as the challenges posed by distribution shifts in structural health monitoring scenarios [33,34]. In particular, those studies explored layer-wise relevance propagation on synthetic aperture radar (SAR) images [31], adversarial training of CNNs on micro-Doppler radar data [30] and the integration of explainable AI into CNNs to improve resilience of classifiers [32]. Another recent work includes adversarial training and domain-invariant feature learning with neural networks to manage distribution shifts in nonstationary time series data [33]. Shu et al. [34] trained a ResNet-34 with learning without forgetting, a continual learning method, to improve the damage classification on concrete structures.

Although our preliminary studies [11,23] only covered small datasets or aspects of the task at hand, they also assumed reliable learning behavior based on high classification f1-scores. Since neural networks are prone to learn semantically irrelevant patterns for certain classes, such as background noise, a critical examination of the learning process is required. Therefore, the novelty of this work is to investigate the robustness and decision-making process of CNNs that are trained with radar data to classify operational wind turbine blades.

With our unique set of radar measurements, we provide several novel contributions to the field of TRM in the context of wind energy applications: (i) Every individual rotor blade holds a number of characteristic structural features revealed by the radar sensor, which can be used to distinguish rotor blades from the same wind turbine with neural networks. (ii) Those unique features of a rotor blade are not invariant to changing environmental and operational conditions (EOCs), suggesting the need for robustly designed models. (iii) Pixel-level augmentation methods, such as blur, reveal the necessity of low-level information for a precise rotor blade classification. (iv) Differently structured neural networks have distinct visual explanations. (v) Higher-order reflections (echoes) that occur in some of the radar measurements can be exploited by the model to detect one of the three blades, which harms the robustness of the model if the other rotor blades are still recognized by the first-order reflection. (vi) Customized cropping helps the model focus on the first-order reflection, suggesting that adaptive cropping or background removal techniques are expected to improve robustness.

Section 2 covers the dataset acquisition and properties, the characteristics of the physical experiment and the theory of neural networks and respective visualization methods. Section 3 is a structured series of machine learning studies to investigate various properties of the radar data and the used neural networks. It begins with the transferability of data from one turbine to another turbine, and continues with the model’s robustness across various EOCs, followed by visual explanations for two neural network architectures revealing model decisions. Section 3 ends with a compact hypothesis that explains the physical and theoretical foundation to classify moving wind turbine blades with FMCW radar data. Finally, Section 4 provides the conclusion of this work.

## 2. Materials and Methods

### 2.1. Data Acquisition

**Figure 2:**
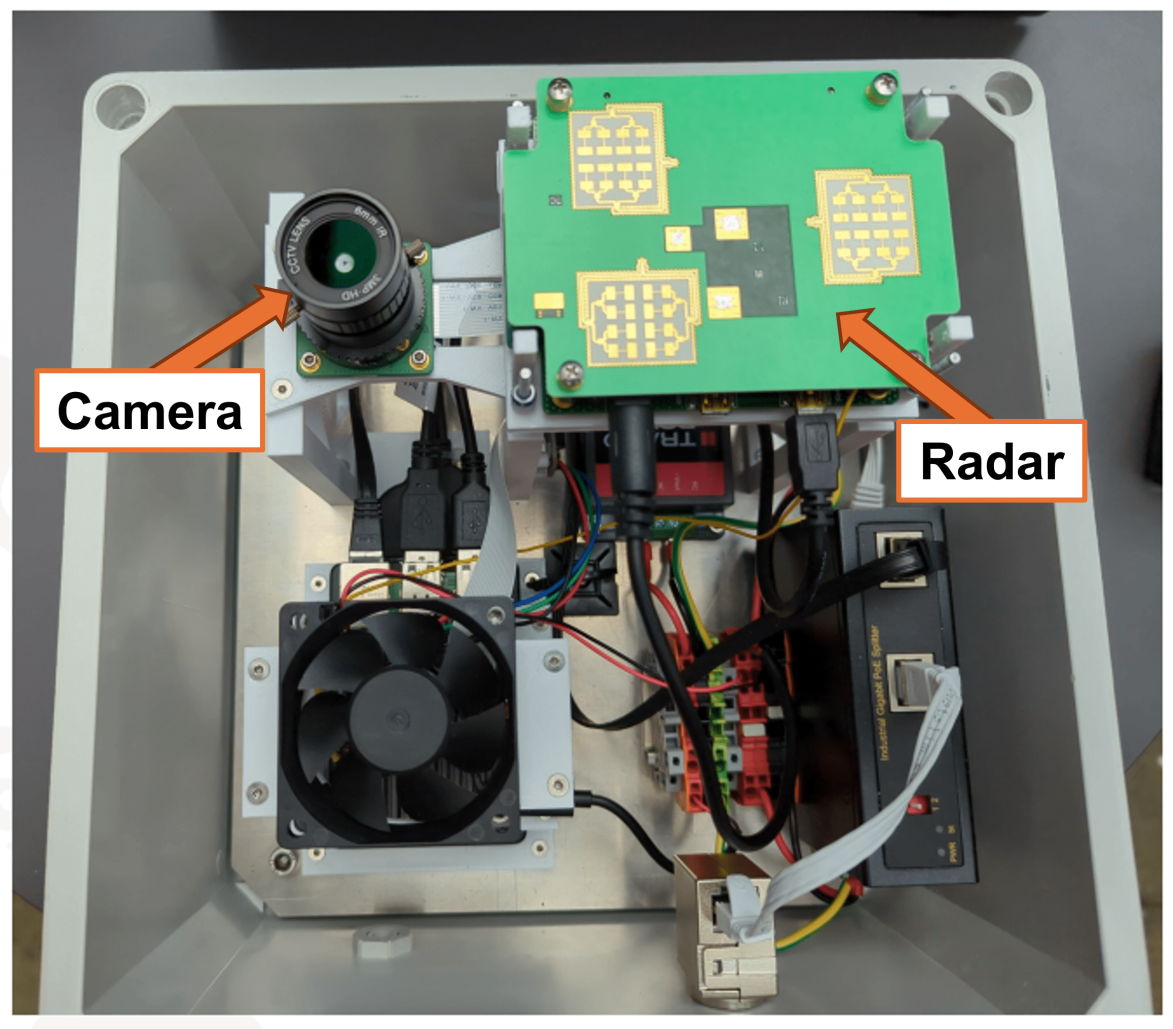
Frequency-modulated continuous wave (FMCW) radar sensor unit [23]. A high-speed camera (100 images/s) is installed next to the FMCW radar (1000 frequency ramps/s). The latter is visible by the green circuit board that is the front-end holding the Rx and Tx antennas.

**Figure 3:**
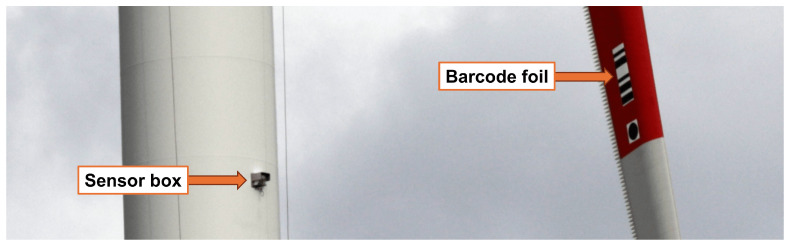
View of the experimental setup [11]. The sensor box is mounted on the wind turbine tower in the main wind direction of 230°. A barcode foil is attached on the surface of the rotor blade facing the sensor box.

For data collection, two equally constructed wind turbines of the same wind park were used (Vestas Wind Systems A/S, Aarhus, Denmark). A 33.4 to 36 GHz FMCW radar (IMST GmbH, Kamp-Lintfort, Germany) and a high-speed camera, placed inside a weatherproof box (Figure 2), were mounted outside of each wind turbine tower at 90 m height (Figure 3). Both boxes were positioned with radar and camera in the main wind direction of approximately 230°. Each rotor blade was equipped with a unique barcode foil on the side facing the sensor box (Figure 3). Every rotor blade pass triggered the radar sensor, which stored the measurements from 0.3 s before to 0.5 s after the trigger event, which forms a single radargram. The radar also simultaneously triggered the camera to store a video for the same interval. From each video, a barcode scanner extracted the ground-truth label for the present rotor blade. An additional setup to collect supervisory control and data acquisition (SCADA) data was also used to store wind parameters for monitoring purposes during each rotor blade pass.

### 2.2. Properties of the Dataset

The current article deals with radargrams collected from 2022 to 2024 on two wind turbines during regular operation. A volume of 276,000 radargrams was measured for the wind turbine “vb06” between 21 October 2022 and 23 March 2024 and 59,000 for the wind turbine “vb07” collected from 15 December 2022 to 25 February 2023. The lower number of examples for “vb07” was caused by an initial delay of the installation and a few technical interruptions that caused shortages in measurement times.

**Figure 4:**
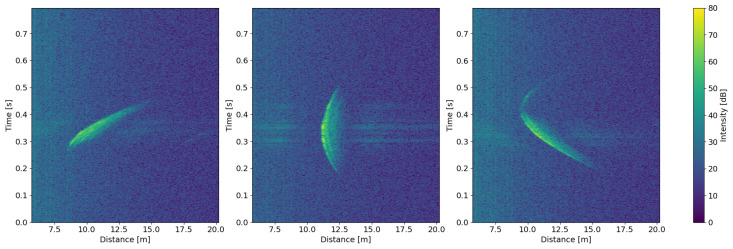
Comparison of radargrams of different EOCs [23]. (**Left**): Nacelle Orientation = 198°, Rotation Speed = 11 rpm. (**Middle**): Nacelle Orientation = 227°, Rotation Speed = 6 rpm. (**Right**): Nacelle Orientation = 256°, Rotation Speed = 10.5 rpm (rpm—rotations per minute).

In general, a radargram X with pixel values $X_{ij}$ constitutes a space-time representation of physical events, i.e., a rotor blade passing the wind turbine mast (Figure 4). In such a radargram, the horizontal axis represents the spatial dimension (range), and the vertical axis indicates the temporal dimension (slow-time). More specifically, the horizontal axis constitutes the distance the reflected signal travels back to the sensor. The vertical axis is the moment in time after a ramp was emitted. Each point in the radargram represents the integrated reflection signal over a given range bin at a specific emission time. Each radargram therefore compresses a rotor-blade pass across the sensor field into a two-dimensional image.

The plots in Figure 4 represent the input format of the radargrams used in this work. The choice of measurement intervals was empirical with the intention of obtaining well-centered rotor blade passes. The time interval ranges from 0.3 s before the event trigger to 0.5 s after the event trigger. With a sweep rate of approximately 1000 frequency ramps/s; these 0.8 s equal 795 ramps in each radargram. For the range interval, the range bins 100 to 350 were selected. With a bandwidth of 33.4 to 36 GHz, a range bin has a resolution of 5.77 cm. This precisely corresponds to a coverage between 5.77 m and 20.18 m. The final size of a radargram is then 795 (ramps) × 251 (range bins).

In accordance with the preliminary works [11,23], the use of slow-time range plots was chosen over range-Doppler maps. While both forms of display are highly related [35], since the range-Doppler map is obtained by a fast Fourier transform (FFT) along the slow-time dimension, they serve different purposes. A slow-time range representation preserves time-domain features such as duration, slope and curvature of the object’s motion over multiple scatter points (Figure 4). These reflected signals over the full motion are a compressed form of blade-specific structural information and are crucial for rotor blade discrimination with neural networks (see Section 3.5). Such characteristic temporal information is averaged out in a range-Doppler map, making it less effective for blade classification.

Applying a further FFT along the range dimension would result in a 2D FFT representation that removed explicit spatial locality in both dimensions. Using either of the two discarded representations as input for a CNN makes the interpretation of visual attributions less intuitive. Another common representation of radar signals is the continuous wavelet transform. The continuous wavelet transform provides time-frequency localization by projecting the measured signal onto predefined wavelet functions at multiple scales. While this approach can be beneficial to reveal characteristic oscillatory components that may aid classification [36], it introduces additional design choices that require prior assumptions. Inappropriate adjustments may suppress or distort structural features relevant for classification. The intention was therefore to preserve the physically meaningful slow-time range representation and rely on the CNN’s ability to learn task-specific filters directly from the data. Prior work has shown that deep CNNs trained on time-domain signals can develop localized frequency-selective filters when respective features are relevant for the task [37].

#### 2.2.1. Problem Geometry

**Figure 5:**
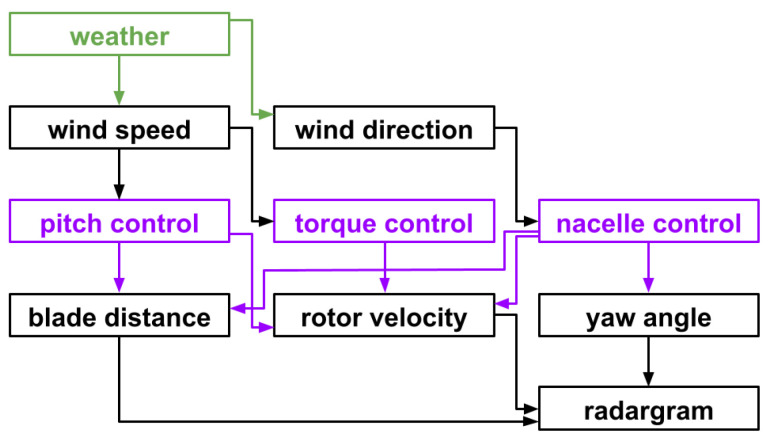
Simplified logical structure of how environmental conditions correlate with a wind turbine’s functioning that shapes the final radargram. Wind conditions, defined by the weather, drive operational adjustments of the turbine which ultimately define how the rotor blade moves in relation to the sensor field. Coloring of control mechanisms (purple) is in contrast to measurements (black).

When a wind turbine is operating, there are several environmental and operational conditions (EOCs) that can change during its deployment. Some of those directly impact others, which creates correlations between certain pairs of EOCs. With an automatic yaw control system, the wind direction and nacelle orientation are similar but not exactly matching. The combination of pitch angle, wind direction, and wind speed defines the resulting rotation speed for a fixed nacelle orientation. In case of fixed pitch angle, wind direction, and nacelle orientation, the only wind parameter that drives the rotation speed is the wind speed. Based on typical control systems applied in wind turbines [38], an illustration of dependencies is given in Figure 5.

Depending on the current EOCs, a multitude of reflection patterns emerge within the radargrams. A variation is shown in Figure 4. An increased rotor speed leads to a reduced time span of the blade in the illuminated volume, creating a shorter profile along the time axis. Different nacelle orientations imply an altered yaw angle that leads to a tilted reflection pattern. Additionally, a pitch angle introduces a slight shift in distance and a change in the width of the reflection pattern.

#### 2.2.2. Fundamentals of Frequency-Modulated Continuous Waves (FMCW)

In this work, the signal transmitted from the FMCW radar is a continuous wave that varies in frequency over time,

(1) $$s_{t}\left(t\right)=\cos\left(2\pi \cdot f\left(t\right)\cdot t\right)\;\;\;\mathrm{with}\;\;\;f\left(t\right)=f_{0}+\theta \cdot t\;\;\;\mathrm{and}\;\;\;\theta =\frac{f_{1}-f_{0}}{\tau }$$

where zero phase, unit amplitude, and a linear frequency sweep are assumed, where the latter possesses a rate θ and ends after duration τ at frequency $f_{1}$.

When the transmitted energy encounters an object, it is in part reflected back. The delay in traveling to the object and back reads $t_{d}=\frac{2D}{c}$ where *D* designates the distance to the object and *c* the speed of light. The frequency of the received signal can be expressed as $f_{r}\left(t\right)=f\left(t-t_{d}\right)=f_{0}+\theta \left(t-t_{d}\right)$ with the so-called beat frequency given by $f_{b}=f\left(t\right)-f_{r}\left(t\right)=\theta \cdot t_{d}$. Substituting and rearranging yields the object distance $D=\frac{c}{2\theta }f_{b}$. Contributions from noise and interference effects were omitted for simplicity. A more detailed mathematical form can be found in [35,39].

#### 2.2.3. Scattering

**Figure 6:**
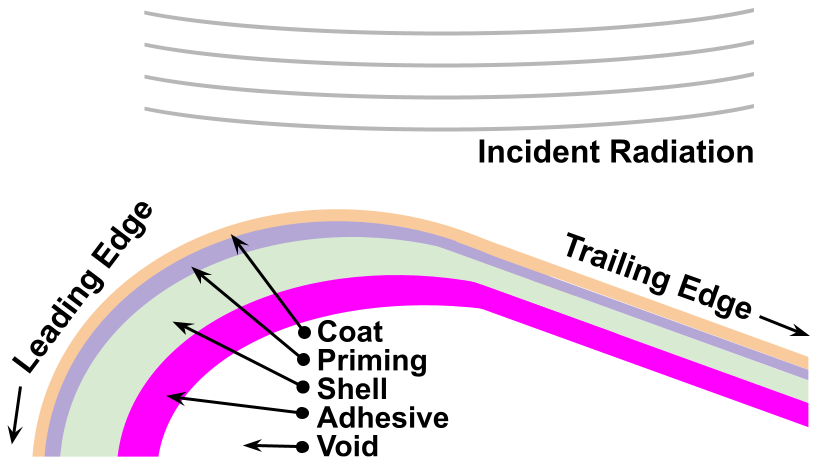
Illustrating the hypothesis behind blade classification. Top-view at (sub-)centimeter radar emissions interacting with the layered internal structure of the radar-facing portion of a wind turbine rotor blade (cross-section shown).

A radiation of 35 GHz has roughly a wavelength λ≈1 cm. The emitted electromagnetic waves of the given frequency partially penetrate the rotor blade, revealing internal structural information [40] compared to optical methods. The exact internal structure will vary between manufacturers and the type of blade [41], however, an illustration of millimeter-scale layering is depicted in Figure 6. The assumption behind the recognition of the three individual blades per turbine is the following: Manufacturing uncertainties (e.g., resin curing or fiber inconsistencies), as well as aging effects (due to transport, assembly, or environmental degradation), will lead to distinct structural fingerprints. They can be learned during training and identified at the test time.

The barcode foil attached to each rotor blade for the optical labeling has no measurable signature in the radar profile, i.e., it had no significant impact on the CNN’s decision. This statement is supported by the results from Alipek et al. [23] where an equally large microwave absorber foil on an identically built wind turbine was shown not to impact CNN classification performance. The microwave absorber foil was also thicker and had better reflective properties. Furthermore, Moll et al. [21] demonstrated the ability of a tower radar sensor with the same frequency level and range to detect small structural defects on rotating objects made with similar fiber composite.

### 2.3. Convolutional Classifiers

Computer vision represents the realm in which deep learning has historically excelled in numerous tasks. Deep learning triggered a revolution, marked by the 2012 ImageNet challenge [42] where a data-driven approach called convolutional neural network (CNN) outperformed conventional hand-made features. In particular, the introduction of ResNet’s skip connections [43] allowed users to train increasingly deeper networks with respective improved performance.

In this paper, rotor blade recognition is treated, which represents a typical computer vision classification problem. Here, at test time, an ML model ϕ is fed with a radargram X to predict the correct class among the K=3 classes indexed by *m*. Its prediction consists of the class with the highest score $\phi \left(X\right)={\mathrm{argmax}}_{m}z_{m}$. The layered, multi-channel CNN architecture specifically entails activation maps $A_{ijkl}$. Here, *k* denotes the channel of layer *l* and pixels in the *x*- and *y*-direction are indicated by i,j.

In order to obtain the trained network ϕ via deep learning, a cross-entropy loss $L_{q}$ is minimized over ground-truth labels $y_{m}\in \left\{0,1\right\}$ for the input radargram $X_{q}$ with $z_{p}$ as its raw logits in the softmax function σ:

(2) $$L_{q}=-\sum_{p=1}^{K}y_{p}\cdot \sigma \left(z_{p}\right)\;\;\;\mathrm{with}\;\;\;\sigma \left(z_{p}\right)=\frac{e^{z_{p}}}{\sum_{m=1}^{K}e^{z_{m}}}$$

In this work, a short custom CNN as well as a ResNet-18 are utilized. The custom CNN has a feature extractor containing three convolutional layers with an intermediate rectified linear activation function (ReLU) and one max pooling operation (MaxPool), followed by two fully connected layers for the classification head. All three convolutional layers have a kernel size of 3 × 3 and stride and padding of 1. The max pooling operation uses a 2 × 2 kernel with a stride of 2. The ResNet-18 architecture was implemented via the Huggingface timm library [44] (version 1.0.9). The utilized model is found under the name “resnet18.tv_in1k”. Its first module holds a convolutional layer with a kernel size of 7 × 7, a stride of 2, and padding of 3, followed by BatchNorm, ReLU, and MaxPool. The remaining modules have convolutions with a kernel size 3 × 3 and a stride and padding of 1 or 2. For training, the ADAM optimizer was deployed with a learning rate of 0.0001 and batch size 16. Smaller batch sizes such as this were shown to improve model generalization and to enable stable training across a wide range of learning rates [45]. The only architectural modification was to define a customized input configuration function inside of the timm library that changed the following parameters: number of classes from 1000 to 3, input size from [3, 224, 224] to [1, 795, 251], and the cropping percentage from 0.875 to 1, because the cropping was applied outside of this library. The ResNet-18 model was, with one exception, also trained from scratch and did not use the pretrained weights from the ImageNet-1k dataset. A larger ablation study with pretrained models is not included in order to keep the analysis focused on the intrinsic properties of the proposed dataset and to ensure interpretability of the presented results. Since variations in pretraining datasets may influence model behavior differently, the primary goal is to assess the direct potential and limitations of the radargram dataset for the given classification task. Secondarily, the model-related motivation is to compare a small, simple CNN with a second CNN that has a significantly larger receptive field and several more nonlinearities. While the 7 × 7 convolution in the first layer is inefficient by modern architectural standards, it does not affect the outcome of the conducted studies. The core architecture of the ResNet-18 is maintained for convenient usage of the timm library and comparability.

### 2.4. Visual Explanations

In an effort to make models more transparent by highlighting relevant characteristics of the input data that contribute to predictions, visual explanations encompass techniques (such as DeconvNets [46]) to provide such insights into the decision-making process of ML models in computer vision. Other visual explanation models followed that do not require architectural change compared to DeconvNets.

One similar method, called Guided Backpropagation [47], produces very sharp, pixel-level saliency maps that highlight detailed edges and textures. While this algorithm is simple and fast, it is not considered a true attribution method based on the axiomatic attribution theory [48], which may lead to partially incomplete attributions in theory. Another popular visual explanation model in computer vision, named GradCAM [49], satisfies the expectations of such a true attribution method. GradCAM by itself provides relevant class-discriminative spatial maps, but which are only coarse, and of low resolution.

The element-wise multiplication of both methods, Guided Backpropagation and GradCAM, leads to high-resolution, class-specific saliency maps and is known as GuidedGradCAM [49,50]. The formula $X^{\mathrm{GuidedGradCAM}}=X^{\mathrm{GuidedBackprop}}⊙X^{\mathrm{GradCAM}}$ combines the advantageous properties of fast and simple high-resolution activations from Guided Backpropagation with the class-discriminative attributions from GradCAM, while mitigating the potential inconsistency of Guided Backpropagation. GradCAM works as follows:

- Identify the target layer (usually the final convolutions l=L), which should both capture high-level features while retaining spatial information,
- compute the gradients of the score *z* for the target class with respect to the *k*-th feature map of layer *L*,
- average gradients to obtain importance of each feature map $\alpha_{k}\propto \sum_{ij}\frac{\partial z}{\partial A_{ijkL}}$ (where the normalization constant is omitted for simplicity),
- weight feature maps to create a coarse localization highlighting influential regions $X_{ij}^{\mathrm{GradCAM}}=\mathrm{ReLU}\left(\sum_{k}\left(\alpha_{k}\cdot A_{ijkL}\right)\right)=\max\left(\sum_{k}\left(\alpha_{k}\cdot A_{ijkL}\right),0\right)$
- upsample heatmap for overlay to original radargram or combination with results from Guided Backpropagation.

Here, Guided Backpropagation modifies the standard backpropagation algorithm in neural networks to allow only a positive forward flow and backward flow of gradients, which helps to highlight the features that are most relevant to the model’s predictions. This is performed by setting the negative contributions to zero [47]:

(3) $$\max\left(\frac{\partial A_{L}}{\partial A_{l}},0\right)\cdot \max\left(A_{l},0\right)$$

Although the traditional variant of GradCAM uses a ReLU to remove negative contributions, turning off the final ReLU also enables visualization of negative attributions. In this way, GuidedGradCAM can highlight both the positive and negative impact of features in the image that only contribute to the decision of a chosen class. GuidedGradCAM reveals which patterns in an input image have contributed to the models’ decision in favor of or against each specific class. Hence, each class (i.e., individual rotor blade) can be evaluated separately.

There is a notable limitation to the combination of Guided Backpropagation and GradCAM. Guided Backpropagation depends on the architecture using ReLU as its nonlinear activation function. Other activation functions, such as sigmoid or tanh, are not compatible with this algorithm [47]. Nevertheless, this limitation does not significantly harm the practical applicability of Guided Backpropagation and therefore GuidedGradCAM, because the ReLU is theoretically well-suited and empirically the most widely used activation function for CNNs [51,52].

## 3. Results and Discussion

In principle, the aforementioned field of tower-radar monitoring (TRM) encompasses several diverse machine learning tasks. In particular, the following section focuses on the fine-grained task of recognizing the specifics of individual rotor blades.

Hereafter, radargram data for all three rotor blades (ROT1 to ROT3) of wind turbines will be considered where studies are conducted by varying important influencing factors. Different power plant sites (i.e., comparable turbines but at adjacent locations dubbed “vb06” and “vb07”) are treated as well as distinct EOCs (rotor speed, nacelle rotation, and pitch angles) and distinct data augmentations of the radargrams. In total, two different machine learning classifiers (custom CNN and ResNet-18) are studied, and two different kinds of results are discussed: quantitative classification performance (as measured by F1 scores) as well as qualitative evaluation of visual explanations (via GuidedGradCAM).

### 3.1. Classifier Robustness Across Turbine Locations

In this subsection, the quantitative classification performance is studied for three scenarios (i.e., static, ad hoc transfer and fine-tuning) where data is available from two equally built turbines, which are located only 300 m apart. Such population-based settings represent a common case in modern structural monitoring applications [53]. To fairly compare the varying performance of the model for the three scenarios, the same partitions were used for each task. A stable, simultaneous measurement period between 15 December 2022 and 25 February 2023 provided either 54,000 or 59,000 rotor blade passes for a turbine and either 18,000 or 19,600 measurements per rotor blade.

#### 3.1.1. Identically Distributed Train and Test Data

A first step in the behavioral and performance analysis of a classification model represents the traditional procedure, with model training and testing performed on two random partitions of the same static dataset. Here, the resulting random train and test splits are therefore identically distributed. This first study evaluates the model’s ability to handle unknown samples from the same feature distribution (i.e., same turbine, same EOCs). For this, the custom CNN (see Section 2.3) was trained and tested separately on both turbine datasets, vb06 and vb07 (see the results in column 1 and column 2 in Table 1).

The performance of the model on turbine vb06 becomes close to a perfect fit, while the data for vb07 seem more difficult to learn. A closer look at the distributions of vb06 and vb07 reveals that rotation speeds below 5 rpm (rotations per minute) are below 40 samples per class (i.e., rare cases). Almost all rotation speeds between 5 and 14 rpm are below 50 samples per class for vb06. Only 6.2–6.4 rpm and 12.2 to 13.8 rpm are above 700 samples per class for vb06. In comparison, vb07 has more than 700 samples per class for 5.6–5.8 rpm and 11.3 to 12.2 rpm. Therefore, the peaks in rotation speed are slightly shifted between both turbines. The other main difference is that vb07 has three times more measurements for underrepresented rotation speeds between 5 and 14 rpm i.e., around 130 samples per class.

With each measurement being a combination of EOCs with many degrees of freedom, there is a large bandwidth of radargram variations for a single rotation speed value. The assumption is that the model fails to correctly learn the underrepresented radargrams for both datasets. Only in the case of vb07 the number of (still) underrepresented examples contributes to more misclassifications in total, which leads to a reduced F1 score.

The complete dataset for vb06 with over 276,000 samples, is much more evenly distributed and contains at least 700 samples per class for all rotation speeds between 5 and 14 rpm. The small custom CNN trained with this dataset reached class-based F1 scores of about 0.95 and proved a solid discriminative ability toward the classification of each rotor blade. Based on this initial study, machine learning experiments beyond Section 3.1 will solely use data from vb06.

#### 3.1.2. Re-Using Trained Classifiers for Other Similar Turbine

Practical scenarios commonly call for reusing a trained classifier in further comparable power plants. Next, a simple transfer learning check was performed by evaluating each of the two models on the test data from the other turbine. Since the core assumption of TRM rotor classifications consists of the uniqueness of each blade (compare Section 2.2.3), low accuracy is foreseen for this ad hoc check. However, the learned representation might be successfully reused with some additional effort (i.e., fine-tuning). For the sake of consistency, the blades in vb06 and vb07 are both denoted as ROT1 to ROT3.

Consequently, column 3 and column 4 in Table 1 display the expected poor performance of that ad hoc check. For comparison, random guessing (or “pure chance”) for three classes in a balanced dataset would lead to a class-based F1 score of 0.333. However, with one class (ROT1) at 0.193 in column 3, and one class (ROT2) at 0.452, there is one result below random guessing and one above. A similar pattern is found for column 4. Such results below random chance can be described as “systematic mistaking” and imply that the model is not only guessing wrong but actively and consistently biasing its errors towards incorrect classes.

**Figure 7:**
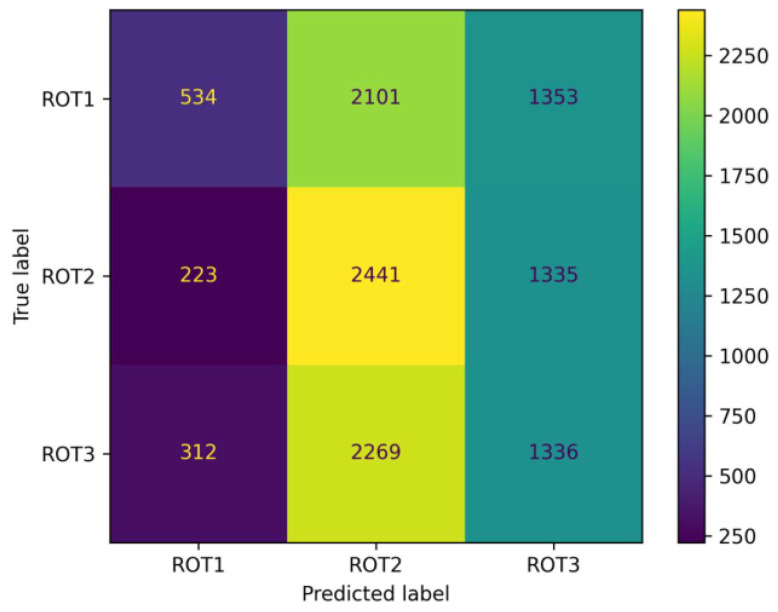
Confusion matrix for a model trained on vb06 data and tested on vb07 data (see column 3 in Table 1).

**Figure 8:**
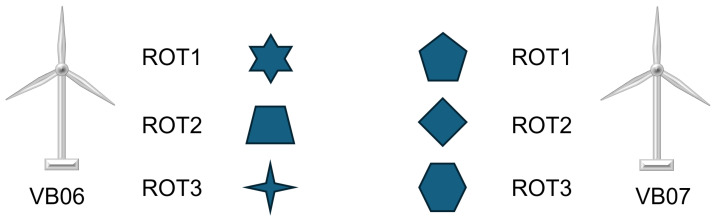
Each rotor blade of both wind turbines is represented by a geometric shape, which symbolizes the unique set of patterns each rotor blade can hold. This symbolic comparison specifically resembles the case of column 3 in Table 1. As retrieved from the confusion matrix in Figure 7 the rotor blades from vb07 have the highest similarity to ROT2 from vb06. This is symbolized by the trapezoid, which is more similar to the forms of vb06.

In general, this observation of poor transfer performance represents another indicator (in addition to Section 3.1.1) that each rotor blade appears to be individual (or even unique) at a level that is measurable by the radar system. Moreover, the confusion matrix given in Figure 7 provides a closer look at the results in column 3 of Table 1. It gives an overview of the predictions when the model trained with vb06 data is evaluated on the vb07 test dataset. A clear trend is visible when comparing the middle column with the other two. Most samples from vb07 are predicted as class ROT2 of vb06. This means that most samples from vb07 have a greater similarity to ROT2 from vb06 than to ROT1 and ROT3. Furthermore, the predictions of the middle column are almost evenly distributed between the three rotors of vb07. This indicates that all three rotor blades of turbine vb07 have a statistically equal similarity to the rotor blade ROT2 of vb06 (see Figure 8 for an illustrative depiction).

Thus, a key question arises: On which learned features does the classifier base its decisions? Hence, in the later parts of this paper, visual explanations will be investigated.

Apart from the aforementioned ad hoc check, a more realistic and widely used approach employs fine-tuning. This fine-tuning approach was performed in two variations. The first variant was extended into a two-stage joint training, where the model is optimized first with one turbine and then with the complete training partition of the second turbine i.e., 60% of the total dataset (Table 1, columns 5 & 6). The second run represents the conventional way, where a small fine-tuning dataset is used, which is 10% of the complete dataset of the second turbine (Table 1, columns 7 & 8).

Regarding the first fine-tuning scenarios, the model that was trained with both turbine training datasets achieves test performances close to the model without pretraining (compare column 1 with 6 or column 2 with 5 in Table 1). However, if the model is pretrained in this way on the training partition of one turbine and subsequently fine-tuned on the complete training partition of the other turbine until saturation, a residual bias toward features of the pretraining turbine remains. These features can have similarities to all classes of the second turbine, which explains the measurable reduction of the maximum F1 score for all three rotor blades. If the model otherwise managed to learn the three rotor blades of each turbine having a high but reduced baseline performance for both turbine datasets, this reduction is still an indicator of increased uncertainty between the three nominal classes, since pairs of different rotor blades are mapped into single classes.

To further analyze this behavior, a conventional fine-tuning run was conducted. When comparing column 7 with 5 or columns 8 with 6 in Table 1, the test performance was significantly lower if the model was fine-tuned with only 10% of the complete dataset instead of the previous 60%. These expected results can plausibly be explained by a larger number of underrepresented EOC configurations and smaller average counts of examples for those underrepresented cases resulting from the smaller fine-tuning dataset.

Since rotor blades can be very unique in the fine-granulated scale of the fiber mantle and the inner blade skeleton, and the radar is able to partially penetrate the material to also measure subjacent structural information [21,40], the radar profile of each rotor blade is inherently different from the others. The presence of structural differences between equally manufactured rotor blades is further supported by measurable mass imbalances within individual blades and mass differences between the rotor blades of the same turbine [54,55]. Therefore, each rotor blade must be treated as another object in the scope of this work (more in Section 3.5).

The observed “systematic mistaking” in the ad-hoc transfer learning study indicates that features learned from one turbine are only partially present in the data of another turbine (Table 1, columns 3 & 4). In particular, the data augmentation study (Section 3.3.4) shows that correct blade discrimination relies on small, persistent scatter patterns located within the crescent-shaped structure of the radargram. Since these low-level features differ between turbines (Section 3.5), the classifier tends to rely on larger and more generic structures. This leads to consistent confusion between rotor blades, as observed in the ad-hoc transfer case. The extreme case is observed when a model is pretrained on one turbine and subsequently fine-tuned with the complete training partition of another turbine. As recently mentioned, it further shows that residual features from the pretraining turbine can introduce bias and additional uncertainty, resulting in reduced maximum F1 scores (Table 1, columns 5 & 6). Therefore, the conducted studies do not indicate the presence of useful transferable representations across different turbines.

### 3.2. Classifier Robustness Across EOCs

In Section 3.1, it was already indicated that each rotor blade can cause individual reflection profiles for the same radar sensor, making each rotor blade distinguishable from other rotor blades of the same wind turbine. A follow-up question would be whether such characteristic features of a specific rotor blade are also invariant to changing EOCs, which is investigated in this subsection with respect to the measurements of one wind turbine, namely vb06.

The focus is on the three wind parameters: rotation speed, nacelle orientation, and pitch angle. Each wind parameter study was conducted in the same way. The complete dataset of vb06 was divided into three partitions with respect to the value of the wind parameter. One partition contains only samples with lower values, another partition has higher values, and the third partition contains measurements with intermediate values between the limits of the first and second partitions. The custom intervals of the different parameters are carefully selected by hand to minimize the impact of their correlation with the inspected parameter. Each partition initially stored the samples sorted by their timestamp. After a deterministic shuffle along the timestamps via numpy random seed, the partition was further split by a 60:20:20 ratio into training, validation, and testing data. Therefore, all three sub-partitions have matching EOCs and a similar feature distribution.

There are other validation methods that closer match real-world scenarios, such as rolling time windows or timely holdout. However, the variation in wind parameters between consecutive months, weeks, or even days can be quite strong, which makes it systematically difficult to determine the contribution of individual wind conditions to the change in classification performance. By previously inspecting the distribution of EOCs and intentionally selecting intervals to minimize correlation, it granted much more control over the studies conducted and facilitated interpretation.

#### 3.2.1. Rotation Speed

The classification results for the rotation speed are given in Table 2. Column 1 shows the model trained and tested with data from an interval with slower rotation speeds (3500+ training samples per class). Column 2 is set up similarly, but for high rotation speeds (2500+ training samples per class). Both columns show high performance for new data from the same feature distribution, i.e., same EOCs. The fifth column shows the performance of the model when trained on both intervals and tested on unseen samples from both trained intervals. The sampling ratio here is balanced for both intervals. This scenario shows a solid fitting result on the data that yields a high F1 score.

When comparing columns 1 and 2 of Table 2, the performance for the “high” partition is the poorest. Here, the high rotation speed leads to a relatively small crescent in radargrams. The correct recognition of small and tiny objects remains a well-known challenge in computer vision and has become a dedicated research subfield [56,57]. Small objects (i) are more prone to perturbations through noise/clutter because of their small visible area, and (ii) make it harder to learn a rich set of meaningful features due to the lack of a nuanced shape or a missing fine texture. Furthermore, (iii) many deep learning models are sensitive to the scale of objects and therefore lack the ability to recognize in small objects their known larger counterparts.

Accordingly, column 3 of Table 2 lists the performance of the CNN that is trained with data from slower rotations and tested on data with higher rotation speed for the same wind turbine vb06. Column 4 is the opposite composition. Both cases of systematic mistaking show that extrapolating to radar measurements of the same wind turbine but for unseen EOCs is not attainable.

Possibly, the classifier can learn diverse features (as proven in previous studies here), but these features are not correlated in a meaningful sense with the appearance of a smaller crescent. Again, the demand arises to understand which learned features the CNN draws on for its predictions. Here, the visual explanations in the later part of the article can help.

Column 6 on the right side of Table 2 represents the interpolation test. Here, interpolation does not refer to the phenomena heavily discussed with regard to overfitting, e.g., in Montanari and Zhong, and Mallinar et al. [58,59]. In the current paper, interpolation means that both intervals, one with slow rotation speeds and one with high rotation speeds, are ingested for training by the same CNN but then the model was tested on samples with rotation speeds between both intervals (900+ testing samples per class). It also differs from mathematical interpolation, since the model is applied to existing radargrams of unknown EOCs and is not attempting to reconstruct missing EOCs or corresponding radargrams. The interpolation results naturally show significantly better performance compared to the extrapolation, but still lack the precision of the regular static setup shown in column 1 and column 2 of Table 2. Results regarding the ResNet-18 are addressed in Section 3.3.2.

To assess the robustness of the reported results, multiple reruns were performed for selected runs summarized in Table 2, including a high-performance case (column 5), an interpolation case (column 6), and a case of systematic misclassification (column 3). Each case was repeated three times using different random seeds for data partitioning and model initialization. The resulting standard deviations ranged from 0.2–0.6% for column 5, approximately 2% for column 6, and 0.4–4.8% for column 3. Similar deviations were found for equivalent runs using ResNet-18. As expected, increasing discrepancies between training and test distributions led to higher variability. However, the magnitude of the observed standard deviations is too small to affect the conclusions of this study. Consequently, averaged results are only reported when statistical exactness is required.

The inference time of the model on an Nvidia RTX 4090 (Nvidia Corporation, Santa Clara, CA, USA) is below 0.5 s per input, and a new radargram is measured and stored every 0.8 s. Sufficient inference time is attainable on edge devices. Thus, real-time processing is technically feasible but not recommended at the current stage, as this design is not yet robust to unknown EOCs. Overall, the above results show that radar measurements of different rotor blades hold some type of characteristic features that define each rotor blade from one another, but those features are partially mutable through changes in EOCs, limiting interpolation and extrapolation. According to these findings, a model for a wind turbine cannot be trained on a fixed dataset, which is collected in a short amount of time, to be robust for all upcoming events. The difficulty is within the high number of degrees of freedom that can alter the characteristic features of each rotor blade under different EOCs. Furthermore, since EOCs such as wind speed and wind direction cannot be controlled and such wind dynamics are not predictable, it makes it highly difficult to collect respective data that covers all potential EOCs of that wind turbine (see Table 3).

At least three possible solutions exist: they either consist of continual learning, where the classifier should be re-trained on recent data that evolves with the altering EOCs, in order to steadily minimize the interpolation or extrapolation gap. Secondly, dedicated augmentations or synthetic data could mimic potential different EOCs, even if they have not yet occurred naturally (see Section 3.3.3). Alternatively, larger and more complex networks [25] are known to generalize better (see Section 3.3.2), but cannot compensate for missing features from unknown EOCs. The impact of changing environmental factors or correlated variables was further discussed in a recent publication with respect to simulated wind turbine data [60].

The hypothesis in Section 3.5, which unifies this work’s results, indicates that latent structural features based on surface and subsurface reflections are characterizing individual rotor blades and enable a classification of those during operation. Aging effects and defects are another form of structural change on and within the rotor blade and are expected to be similarly revealed via radar signal reflections. Those structural alterations happen gradually and subtly change the characteristic features of a rotor blade over time. Such alterations will presumably be unknown to the model, but can be similarly addressed by the previously mentioned continual learning methodology.

#### 3.2.2. Nacelle Orientation and Pitch Angle

The following Table 4 and Table 5 results are obtained by the same procedures previously explained and also lead to similar partition sizes and findings. To save space and avoid redundancy, the respective tables are provided with short remarks about anything different from the first example regarding the rotation speed. Details about the sampled EOCs are provided in Table 6 and Table 7, respectively.

### 3.3. Visual Explanations of Radargrams

The above results indicate the existence of characteristic features for each rotor blade, enabling discrimination between multiple rotor blades. To make more specific statements and build trust in the model, GuidedGradCAM will be used to visualize the dominant features that a CNN has learned to correctly classify three rotor blades of the same wind turbine. For the inference stage during GuidedGradCAM, the batch size was set to 1 in order to compute visual explanations for individual radargrams.

#### 3.3.1. Custom CNN on Original Radargrams

First, GuidedGradCAM attribution evaluations were performed on the last convolution layer of the custom CNN. The attributions were upsampled to match the input size (795 × 251 pixels).

**Figure 9:**
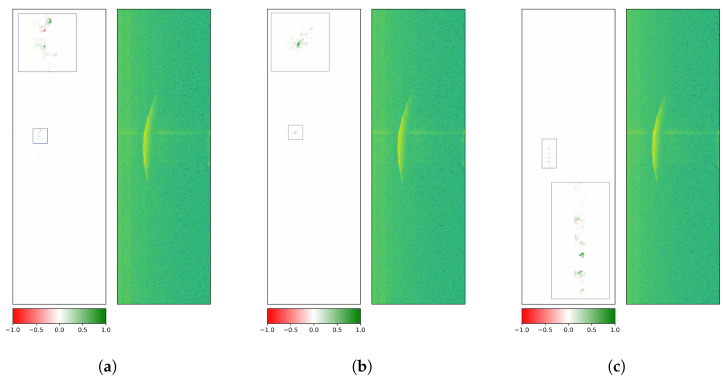
Visualized decisions of the custom convolutional neural network (CNN) for the three classes in the case of the predicted class and given ground truth label. Regions of interest are additionally plotted with upscale factor 4 to highlight small attributions (Case 1). (**a**) Class 1, predicted class 1, ground truth class 1. (**b**) Class 2, predicted class 1, ground truth class 1. (**c**) Class 3, predicted class 1, ground truth class 1.

Figure 9 treats an exemplary radargram of ROT1, which is correctly predicted by the custom CNN: visual explanations for class 1 (correct), classes 2 and 3 (both wrong) are depicted. Since mainly the middle section of the crescent in all three visualizations is activated, the more dominant first reflections on the rotor blade surface seem to be of interest to the model for its decision. Here, positive and negative contributions (i.e., for and against a given class) are located at the core reflection site.

**Figure 10:**
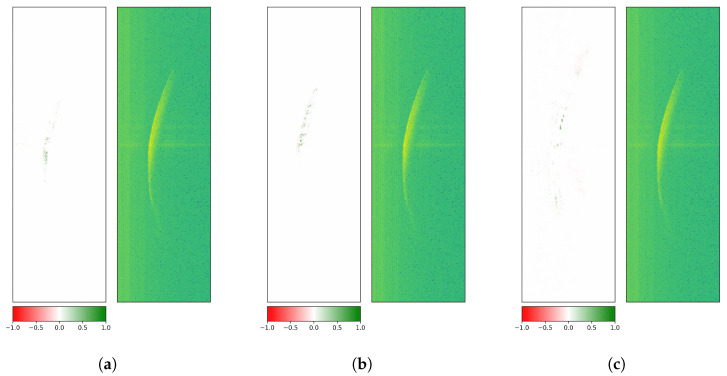
Visualized decisions of the custom CNN for the three classes in case of the predicted class and given ground truth label (Case 2). (**a**) Class 1, predicted class 2, ground truth class 2. (**b**) Class 2, predicted class 2, ground truth class 2. (**c**) Class 3, predicted class 2, ground truth class 2.

Figure 10 covers an illustrative radargram of ROT2, which is accurately classified by the custom CNN: visual explanations for classes 1 (wrong), 2 (correct), and 3 (wrong) are shown. Results still exhibit similar attributions for class 1 and class 2, but a different pattern for class 3: apart from positive (green) contributions at the central part, a significant number of peripheral negative (red) pixels along the crescent are visible.

All investigations had been carried out with three datasets holding measurements with low rotation speeds, high rotation speeds, or low and high rotation speeds, and led to very similar results.

#### 3.3.2. ResNet-18 on Original Radargrams

The above ablation study with the custom CNN showed mainly small, spatially concentrated visual explanations. Keeping the shallowness and size of the custom model in mind, such behavior was likely caused by the small field of view of the final convolutional layers. Therefore, a second deeper model (ResNet-18) was selected to also test a widely known/investigated model architecture. Additionally, the observation that green attributions for each class are observed on the main reflection front, even though the model has correctly classified the radargram could hint towards a weakness of the custom CNN. If reinforcing patterns of all three classes are very closely located on the radargram, and the radargram happens to be slightly distorted (caused by changing EOCs), one may expect the discriminative ability to rapidly decline.

Thus, the last three columns in Table 2 show a robustness comparison regarding the interpolation capacity of the custom CNN and the ResNet-18. Here, the decline in classification performance in the interpolation task is more significant for the custom CNN (as expected). With the ResNet-18 considered the more capable architecture, an additional study using pretrained weights from the ImageNet-1k dataset was conducted to further assess the limitations of the dataset and task. The experiment trained on low and high rotation speeds was first repeated three times with different random seeds with randomly initialized weights. The same configuration and partitions were then used for the same ResNet-18 architecture initialized with pretrained ImageNet-1k weights. The pretrained variant reached training saturation approximately three times faster with a 1–2% higher baseline F1 score when evaluated on test data drawn from the same EOC distribution. This result is notable given that ImageNet-1k consists of natural images and does not contain sensor-specific representations such as radargrams. A common explanation in the transfer-learning literature is that early convolutional layers learn generic feature detectors (i.e., edges, simple geometric structures, and textures) [46] that are transferable across domains [61,62] and provide a more favorable initialization for training convergence [63].

Interpolation performance improved when using the pretrained ResNet-18, as shown in the added column 8 (compared to column 7) in Table 2, indicating increased robustness under previously unseen EOCs. This behavior is consistent with the comparison between the custom CNN and the ResNet-18 trained solely on radargram data, where the more expressive architecture showed better generalization. Overall, the results suggest that more effective feature representations improve robustness and generalization, even when the pretraining domain differs substantially from the target domain.

**Figure 11:**
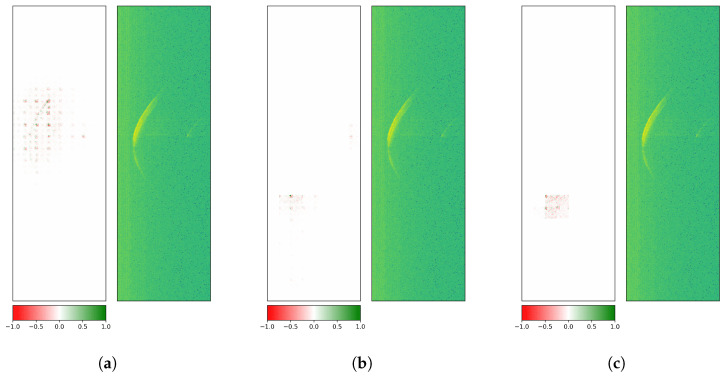
Visualized decisions of the ResNet-18 for the three classes in the case of the predicted class and given ground truth label (Ground truth class 1). (**a**) Class 1, predicted class 1, ground truth class 1. (**b**) Class 2, predicted class 1, ground truth class 1. (**c**) Class 3, predicted class 1, ground truth class 1.

To provide a clearer picture of why ResNet-18 seems more robust, the model’s class attributions were also computed via GuidedGradCAM. Figure 11 generally exhibits activation patterns that are broader than in the case of the custom CNN. Similar observations were found in Selvaraju et al. [49] where a ResNet yields more extended visual explanations than the Visual Geometry Group (VGG) architecture [64]. In Figure 11 the wrong classes (i.e., 2 and 3) interestingly lead to explanations far off the crescent where only noise resides.

**Figure 12:**
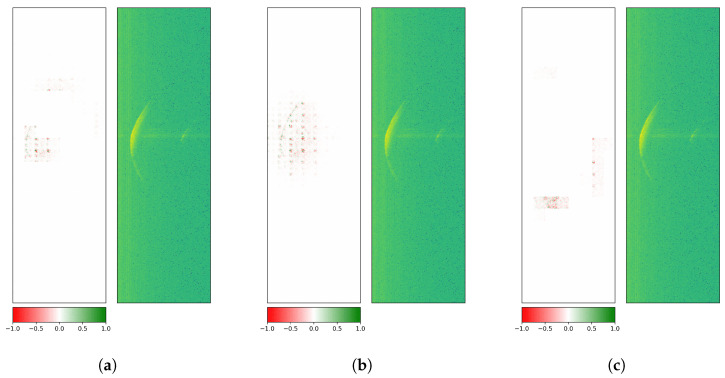
Visualized decisions of the ResNet-18 for the three classes in the case of the predicted class and given ground truth label (Ground truth class 2). (**a**) Class 1, predicted class 2, ground truth class 2. (**b**) Class 2, predicted class 2, ground truth class 2. (**c**) Class 3, predicted class 2, ground truth class 2.

When a radargram contains a second, smaller sickle-shaped reflection on the right-hand side of the first reflection, as in Figure 12, it can influence the decision-making of the model (see wrong class 3). This smaller reflection represents the echo of the radar sensor. It occurs when the first signal sent from the radar is partly reflected by the sensor and reaches the reflecting object a second time. It therefore is of reduced intensity and has traveled twice as far, which explains its location at double the distance.

**Figure 13:**
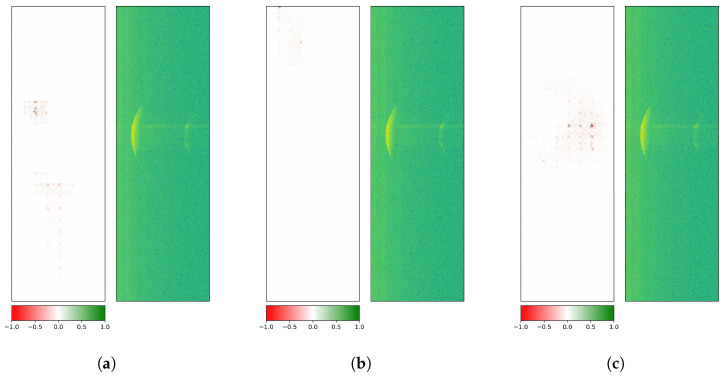
Visualized decisions of the ResNet-18 for the three classes in the case of the predicted class and given ground truth label. (Ground truth class 3). (**a**) Class 1, predicted class 3, ground truth class 3. (**b**) Class 2, predicted class 3, ground truth class 3. (**c**) Class 3, predicted class 3, ground truth class 3.

Analogous behavior can be observed in Figure 13, where the decision on the correct class is based on this second-order effect.

#### 3.3.3. Custom CNN and ResNet-18 on Cropped Radargrams

In the next step, the impact of random cropping was investigated, since models were shown to focus on distant noise patterns (Section 3.3.2.) From the original radargrams (size 795 × 251 pixels), the center section (size 450 × 251 pixels) was first extracted to remove most parts without the actual event. Then, random crops (size 224 × 224) were extracted for model training. The model was trained from scratch twice with the exact same dataset, either by combining cropped or raw radargrams of low and high rotation speeds (Table 8). Random cropping seems to reduce the training and testing performance of the ResNet-18. This means that random cropping makes it more difficult for the model to classify the three rotor blades for data with the same feature distribution. Either previous performance was based on spurious features (i.e., shortcut learning), which cropping has taken away. Alternatively, cropping has been too aggressive and eliminated relevant parts of the crescent.

Interestingly, the interpolation test revealed additional helpful insights. As given in Table 8, random cropping improved the overall ability of the model to correctly classify the rotor blades when it was tested on radargrams with unknown rotation speeds between the intervals used for training. In particular, the identification of class 2 (ROT2) improved significantly using random cropping. This overall increase in the F1 scores may indicate that random cropping supports the model in having a better general understanding of the individual rotor blade reflection and in ignoring (a) local noise patterns around the main reflection front and (b) general background noise before and after the actual event during the complete measurement. This would also explain why the training time until the optimization was saturated was longer for random cropping. This was probably because each radargram was randomly cropped in a different location, making it harder for the model to learn the relevant patterns of each class.

**Figure 14:**
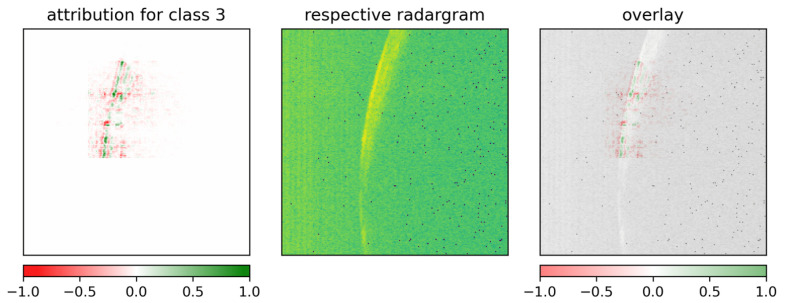
Typical attributions found for a centered cropping in case of a correct classification.

To verify these assumptions, GuidedGradCAM was also applied to the ResNet-18 model trained with randomly cropped radargrams. Figure 14 demonstrates the usual attributions found for random cropping in this model. The training and testing dataset for this cropping example only used radargrams with low rotation speed. The plotted attributions are for class 3 (ROT3) in case of a correct classification of class 3. Compared to correct predictions with no random cropping, the attributions found have a very compact rectangular shape and capture a limited area centered around the main reflection front. This indicates that the model was better conditioned to comprehend the reflection information of the rotor blades themselves rather than the surrounding background noise.

**Figure 15:**
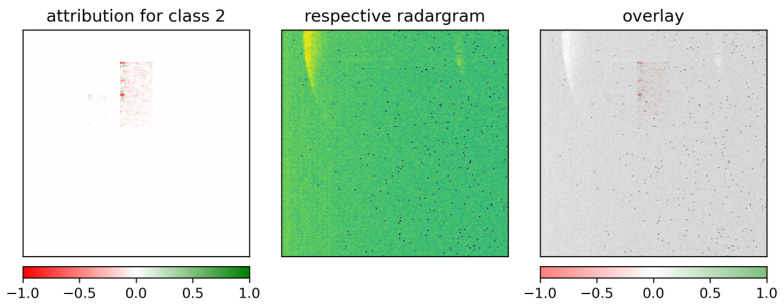
Example of unsuitable attributions from too aggressive cropping.

A less expected example occurred for a ResNet-18 trained with radargrams of low and high rotation speed using the same cropping as in the previous test. In Figure 15, the attributions of class 2 for a correct prediction of the same class exhibit a pattern that contributes to the correct class decision, which is not part of the two implied reflection fronts. This means that the model has learned areas of unwanted background noise to correctly classify the rotor blades. This type of unsuitable learning behavior is most likely caused by too extreme random cropping. With the dimensions of the cropping kept unchanged and added radargrams of high rotation speed having shorter reflections in the vertical, since they move faster through the sensor field, the ResNet-18 was exposed to several examples where the crops do not contain sufficient parts of the actual rotor blade reflection. This must have forced the model to extract meaning from the remaining parts of the images, i.e., the background noise.

Considering the previous observations, random cropping shows potential, but only when applied correctly and fit for the data. This means that one needs to know the dimensions of the expected objects of interest within the images. Since an operational wind turbine has multiple ways to calibrate to wind speed and direction, by nacelle orientation and pitch angle, affecting rotation speed, angle, and distance to the sensor, there are many possible different radargrams. One must also consider that some architectures are not capable of handling multiple resolutions or performing on resolutions other than the training resolution. Models of the latter kind cannot predict new radargrams with the original shape when they were trained on cropped data. Although the approach enhanced the model’s focus on rotor blade reflections and improved interpolation performance, the maximum training and test accuracy slightly declined. This decrease is attributed to the combination of centered and random cropping, which prevented the model from memorizing systematic background noise patterns that otherwise facilitated radargram classification. Traditional random cropping can introduce noise that harms classifier performance as demonstrated in Figure 15, because misplaced crops misalign with regions of interest (ROI), i.e., crescents representing rotor blade reflections. Previous work showed that masking or guiding the cropping procedure can reduce such noise and improve the accuracy in CNN-based classifiers or object detectors [65]. An overview of popular adaptive ROI extraction methods, such as learned region proposals or instance segmentation models that perform ROI cropping and alignment, can be found in Liu et al. [66]. One article specifically focused on a novel ROI extraction layer in CNNs that explicitly learns where to crop based on the image content [67]. While these approaches appear as promising improvements over random cropping, an extensive and systematic study of such learning procedures to maximize model performance is beyond the scope of this work.

#### 3.3.4. ResNet-18 on Augmented Radargrams

**Figure 16:**
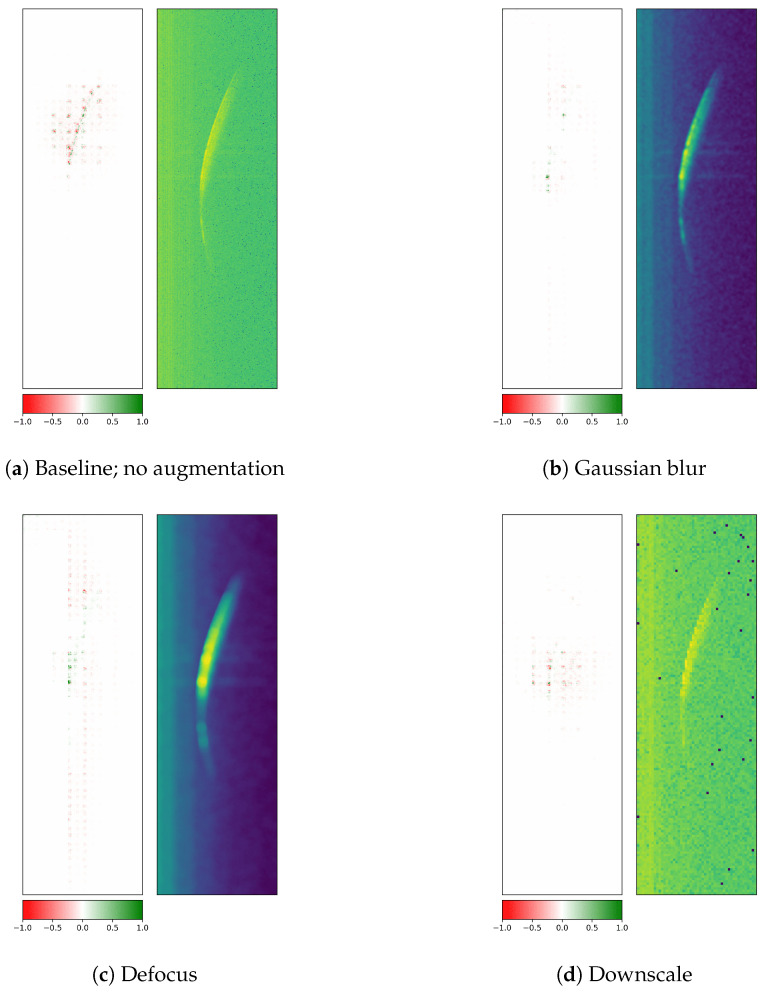
Comparison of pixel-level data augmentation techniques on the same measurement example.

Finally, another way to assess model robustness is to evaluate it on perturbed test data (after training on the original radargrams). The evaluated methods are Gaussian blur, defocus, and downscale and were used from the albumentations library [68] (version 1.4.15) for PyTorch (version 2.1.0 with CUDA version 12.1). The degree of the applied augmentations is chosen to be rather severe to create a more noticeable change in attributions (Figure 16).

The first augmentation technique is Gaussian blur, which applies a Gaussian filter with a random kernel size and a sigma value as the standard deviation of the kernel. By reducing image noise and detail, it creates a smoothing effect. Subfigure (b) shows a change in attributes, with less attention to the middle section of the main reflection front and even less attention to the immediate surrounding area. The class-based test F1 scores on the augmented version of the test data samples are 0.174, 0.509, and 0.0, respectively. This indicates that with all low-level patterns smoothed out, and therefore removed, the model fails to correctly detect each rotor blade. Performance fell below random guessing, with class 2 systematically misclassified as either class 1 or 3.

The defocus method also uses a Gaussian blur technique, but with disc kernels, creating a camera-like smoothing effect. The blur effect in Subfigure (c) is even stronger than the one in Subfigure (b), creating different attributions for the same image and observed class. The computed F1 scores are 0.0, 0.497, and 0.0. displaying a systematic confusion of the model with a 100% bias towards class 2.

The third listed augmentation technique downscales an image to a lower resolution. The downscaled image is then upscaled again, but without recovering the lost information from the downscaling. The image then represents a more pixelated version. The downscaling and upscaling procedure reveals some pixels with failed measurements in Subfigure (d) that have a level of intensity below the background noise, given by the few blue pixels. Similarly to the blurring methods shown previously, the F1 score fell significantly to values of 0.057, 0.255, and 0.491. This method is another variant to simulate a loss of detail. It becomes a regular radargram with lower resolution imitating a radar system with different settings, i.e., wider range bins and lower frequency, which leads to fewer ramps for the same time window and fewer range bins for the same distance interval. Although the attributions are shaped and located similarly to the baseline version (Subfigure (a)), the reduced performance indicates that the model is primed to the radargram settings of the original training data.

The stronger the pixel-level augmentation parameters were set, the worse the overall classification performance of the model, indicating the main dependency of low-level features for a successful rotor blade discrimination. Here, high-level features refer to the shape or orientation of the crescent, and low-level features encompass texture. As the attributions for all tested data augmentation techniques have shown, the main reflection front is still partially detected by the model, but it fails to recover characteristic low-level patterns from the pixel-level manipulated images, which are necessary for the correct classification.

Apparently, some image augmentations, such as downscaling, have an intuitive correspondence to a change in radar configuration. However, a reliable correspondence between radar hardware degradation (e.g., antenna, transmitter/receiver chain, ADC, …) and pixel-level modification in the resulting radargrams is non-trivial. The coupled signal processing within modern radar sensors is too complex, and mapping their degradations to image augmentations and related intensities would be speculative. Studies conducted in this section were intended to perform a stress test on the model’s behavior and the impact on visual explanations, not to model specific physical failure modes.

### 3.4. Impact of Weather Conditions on Radargrams

While wind conditions have a direct impact on the problem geometry, i.e., the movement of the rotor blade relative to the sensor (Section 2.2.1), the weather can also have a systemic influence on the overall radargram. Increased system temperature may occur during hot summer months in Europe and potentially increase thermal noise in the sensor. Thermal noise in the radar receiver was shown to negatively affect the accuracy of range and velocity measurements [69]. Another relevant condition is the impact of rain. In laboratory and field measurements on rain detection [70], the atmospheric attenuation of mild rain was negligible, while heavy rain was expected to have a measurable impact on radar data. Water molecules in the form of raindrops have a higher absorption and scattering of electromagnetic waves at typical microwave and radar frequencies than dry air. However, both conditions are rather critical in applications with small objects tracked at great distances and less problematic in this experimental setup with a single large rotor blade at comparatively short distances (<15 m). Thermal noise increases the overall noise floor, and rain causing attenuation reduces signal strength. Thus, both effects degrade the signal-to-noise ratio (SNR), but they are systemic and statistical, i.e., they affect all measurements similarly. Additionally, CNNs trained on radargrams focus on spatial patterns and relative contrast between pixels, which implicitly compensates for moderate changes in the SNR.

### 3.5. “Structural Fingerprint” Hypothesis

The core hypothesis of this work is that each rotor blade of a wind turbine exhibits persistent characteristic structural differences arising from manufacturing uncertainties and aging effects. Manufacturing variability in particular, is well known for rotor blade production and is largely related to heavy, repetitive manual labor and limited process control [55]. Such variability manifests, among other aspects, in measurable mass imbalances both within individual rotor blades and between the blades of the same turbine [54]. Since radar waves partially penetrate glass fiber composites and are sensitive to their surface and subsurface structural properties, radar-based measurements provide a plausible physical basis for capturing these differences in wind turbine blades [21,40].

Based on the conducted studies, this hypothesis needs to be critically evaluated. A typical radargram, as shown in Figure 4, is a strong compression of a three-dimensional motion into a two-dimensional image. It encodes the reflected signal intensity as a function of slow time (ramp index, y-axis) and distance (range bins, x-axis). The dominant time-varying cause of reflections in this setup is a segment of one rotor blade entering and leaving the sensor field, which represents the pixels of high intensity in the radargram.

Since each pixel is an integrated intensity over the three-dimensional cross-section in that range bin, it effectively is the aggregated reflection of the respective rotor blade segment, i.e., spatial information is lost during that compression. Nevertheless, such a pixel computed by all reflections of that rotor blade segment is observed across many range bins and emission times during a rotor blade pass, which forms a crescent-shaped trajectory in the two-dimensional radargram. Positive attribution maps of a trained CNN show a focus on those crescents for the correct classifications (Figure 12 and Figure 14). Paired with a robust performance across known EOCs (Section 3.1.1), this indicates the existence of reliable blade-specific structural features, despite substantial compression. These features manifest as consistent pixel compositions for given EOCs, which enable the discrimination of individual rotor blades.

Furthermore, moderate variations in the installation of individual rotor blades on the same turbine are unlikely to impact classification performance. For example, if one rotor blade was reinstalled in a slightly stronger tilt than before, relative to the tower and therefore the radar sensor, this would lead to a slight shift in the radar crescent for a given EOC. It would not change the reflective properties and, therefore, it would not affect the model performance as long as comparable shifts were present in the training data across different EOCs. These observations suggest that the composition of reflections in the radargram not only resemble gross geometric motion, but also encodes surface-dependent scattering, characteristic of each individual rotor blade, influenced by material properties, structural layering, and local surface and subjacent imperfections. While such structural variations are assumed to represent the primary physical mechanism underlying the demonstrated discrimination of nominally identical rotor blades via radargrams, establishing a direct correspondence between specific material deviations and radar-derived representations remains challenging and is beyond the scope of this study.

## 4. Conclusions

In order to advance the emerging field of tower-radar monitoring, the robustness and explainability of convolutional classifiers were covered in this paper. It was found that fine-grained features are necessary for rotor-blade classification, which are sensitive to the possibly occurring perturbations. This task of identifying individual rotor blades is a fundamentally different challenge for a CNN than training it to recognize the overall structure or appearance of a turbine blade in contrast to unrelated objects. Using a larger model, i.e., ResNet-18 versus the compact custom CNN, yields some robust performance; however, latency limits and constrained resources could be hindering in real-time real-world systems.

The visual explanations revealed the fixation on second-order reflections of the rotor blade for some classes. These reflections only occur for some EOCs, which makes it less reliable for a general discrimination of the rotor blades. Introducing a subtle cropping based on these findings helped the model to use the main crescent of the radargram for all rotor blades. Deliberately induced data augmentations, such as random crops, helped to boost the interpolation ability. Accordingly, on the basis of what has been attained herewith, a multitude of compelling research avenues exist:

- Thanks to the visual explanations, the high-intensity areas in the radargrams were identified, which are decisive for blade classification. In contrast, the remaining background of the crescent turned out to be obstructive in some instances. Thus, a foreground detection step could be introduced, leading to background removal or context-based adaptive cropping to aid blade recognition.
- In the current work, perturbations were investigated, and the kinship among image transformations was pointed out. Here, augmentations help to enlarge or balance datasets; tower-radar monitoring, as a recent computer vision topic, however, will likely also benefit from the imagery of resembling domains as well as synthetic data [12].
- The intricate but isolated task of recognizing individual rotor blades was tackled. Practical tower-radar monitoring systems nonetheless face multi-task challenges. The growth of wind energy often conflicts with the conservation of wildlife. In order to protect species, wind turbines will not even become approved in sensitive locations, or existing ones have to be temporarily switched off at designated times. In order to resolve this green-green dilemma (climate versus wildlife protection), novel detection capabilities are being developed with respect to birds and bats in the vicinity of wind parks [71,72].
- By addressing the impact of changing EOCs, the fundamentally dynamic setting of tower-radar monitoring was acknowledged. In addition, the continual learning challenge should be treated in which the classifier adapts to new and changing data as it emerges during monitoring. Here, only weak or noisy labels are available [73] and, e.g., regularization [74] must strike a balance between preservation versus flexibility.

## Figures and Tables

**Table 1:** F1 scores on various dataset compositions. **(Column 1)**: the model was trained on the training partition for turbine vb06 and evaluated on another partition of the same turbine. **(Column 2)**: equivalent setup with vb07. **(Column 3 & 4)**: models were each trained on one turbine and evaluated on the test partition of the other. **(Column 5)**: the model was first trained on vb06, then it was fine-tuned on 60% of the complete dataset of vb07 and then evaluated on test data from vb07. **(Column 6)**: represents the vice versa of column 5. **(Column 7)**: same configuration as column 5 but fine-tuned with only 10% of the complete dataset. **(Column 8)**: represents the vice versa of column 7.

Train → Finetune → Test →	vb06 - vb06	vb07 - vb07	vb06 - vb07	vb07 - vb06	vb06 vb07 vb07	vb07 vb06 vb06	vb06 vb07 vb07	vb07 vb06 vb06
ROT1	0.981	0.910	0.193	0.478	0.881	0.968	0.749	0.917
ROT2	0.979	0.898	0.452	0.208	0.863	0.971	0.727	0.896
ROT3	0.980	0.863	0.336	0.217	0.813	0.971	0.716	0.910

**Table 2:** F1 scores for systematic speed variation. **(Columns 1 & 2)**: test case when EOCs match the training data. **(Columns 3 & 4)**: lack of extrapolation for rotation speeds unseen at training. **(Column 5)**: model trained with high and low rotation speed measurements. **(Column 6)**: interpolation performance by excluding intermediate rotation, and therefore, wind speeds. All results are for the custom CNN except the last. **(Column 7)**: ResNet-18 performance from Section 3.3.2 is indicated by an asterisk (*) and provided here for convenient comparison. **(Column 8)**: Same as column 7 but initialized with pretrained weights from ImageNet-1k, which is indicated by a second asterisk (**).

Train → Test →	Low Low	High High	Low High	High Low	Both Both	Both Mid	Both * Mid *	Both ** Mid **
ROT1	0.962	0.929	0.478	0.344	0.970	0.543	0.679	0.868
ROT2	0.965	0.913	0.324	0.398	0.965	0.545	0.657	0.866
ROT3	0.979	0.944	0.007	0.173	0.975	0.635	0.769	0.921

**Table 3:** EOCs for systematic speed variation. For fair comparison all other relevant meta-parameters are reasonably set: e.g., the pitch angle is allowed to vary within a very small interval at same wind speeds and direction, in order to provide a sufficient number of train and test samples.

Meta-Parameter	Low	Mid	High
rotation speed [rpm]	{7.0, 9.0}	{9.5, 11.5}	{12.5, 13.5}
nacelle orientation [°]	{200, 270} *		
pitch angle [°]	{−2.5, −1.5} *		
wind direction [°]	{150, 300} *		
wind speed [m/s]	{4, 7}	{7, 8}	{8, 11.5}

* Same parameter interval for all three partitions.

**Table 4:** F1 scores for systematic nacelle variation with similar findings as in Table 2.

Train → Test →	Low Low	High High	Low High	High Low	Both Both	Both Mid
ROT1	0.926	0.999	0.235	0.430	0.971	0.458
ROT2	0.935	0.991	0.400	0.264	0.971	0.344
ROT3	0.950	0.992	0.291	0.101	0.979	0.538

**Table 5:** F1 scores for systematic pitch angle variation with similar findings as in Table 2.

Train → Test →	Low Low	High High	Low High	High Low	Both Both	Both Mid
ROT1	0.985	0.972	0.503	0.459	0.986	0.889
ROT2	0.985	0.974	0.470	0.082	0.985	0.696
ROT3	0.990	0.973	0.065	0.013	0.986	0.821

**Table 6:** EOCs for systematic nacelle variation. The difference between nacelle orientation and wind direction were matched for each sample to be below ten degrees.

Meta-Parameter	Low	Mid	High
rotation speed [rpm]	{0, 13.5} *		
nacelle orientation [°]	{200, 220}	{225, 245}	{250, 270}
pitch angle [°]	{−5, 2} *		
wind direction [°]	{190, 230}	{225, 245}	{240, 280}
wind speed [m/s]	{0, 10.5} *		

* Same parameter interval for all three partitions.

**Table 7:** EOCs for systematic pitch variation. To reveal the potential impact of the pitch angle, the rotation speed must be kept in a very small interval since it is highly changing the radar profile, potentially inducing a bias. For a fixed wind direction, nacelle orientation, and rotation speed, the wind speed correlates with the pitch angle, because the pitch angle defines how fast the rotor blade would move under the contemporary wind speed. Therefore, if the rotation speed is held fixed, the pitch angle and wind speed cannot be decoupled, but it appears to be the only way to investigate the impact of the pitch angle.

Meta-Parameter	Low	Mid	High
rotation speed [rpm]	{12.25, 13.25} *		
nacelle orientation [°]	{237.5, 262.5} *		
pitch angle [°]	{−3, 3}	{5, 10}	{12, 18}
wind direction [°]	{237.5, 262.5} *		
wind speed [m/s]	{8.25, 11.5}	{10, 16}	{14.5, 18.5}

* Same parameter interval for all three partitions.

**Table 8:** The first six columns show the F1 scores for a ResNet-18 trained and tested on a joint dataset of radargrams with low and high rotation speed. The last three columns list the F1 scores for a ResNet-18 trained with data containing low and high rotation speeds and tested on radargrams with unknown rotation speeds between training data intervals. The first row shows results without data augmentation, and the second row with random cropping.

	Train ROT1	Train ROT2	Train ROT3	Test ROT1	Test ROT2	Test ROT3	Test * ROT1	Test * ROT2	Test * ROT3
raw	0.996	0.996	0.999	0.980	0.976	0.988	0.744	0.677	0.820
crop	0.953	0.953	0.971	0.946	0.946	0.964	0.757	0.814	0.823

* Additional interpolation test.

## Data Availability

Due to confidentiality agreements with the wind power plant operator, the entire dataset supporting the findings of this study cannot be publicly shared. A small subset of the data has been deposited in Zenodo [75] and the source code for this work is publicly available on GitHub https://github.com/SaILaIDiN/TRM-of-Wind-Turbine-Blades-with-CNNs (accessed on 2 February 2026).

## Abbreviations

The following abbreviations are used in this manuscript:

EOC	Environmental and operational condition
FMCW	Frequency-modulated continuous wave
TRM	Tower-radar monitoring
ML	Machine learning
AI	Artificial intelligence
LIME	Local interpretable model-agnostic explanations
UAV	Unmanned aerial vehicle
CNN	Convolutional neural network
SAR	Synthetic aperture radar
SCADA	Supervisory control and data acquisition
FFT	Fast Fourier transform
ReLU	Rectified linear unit
MaxPool	Maximum pooling
ROI	Region of interest
SNR	Signal-to-noise ratio
